# Effects of Berberine, White Mulberry Leaf Extract and Chromium Picolinate on Body Composition and Insulin Resistance in Adults with Obesity: A Randomized Controlled Trial

**DOI:** 10.3390/nu18172801

**Published:** 2026-08-27

**Authors:** Konstantinos Xenos, Konstantinos Chalkias, Giannis Chryssou, Evgenia Alexandrou, Emilia Vassilopoulou

**Affiliations:** 1 Department of Nutrigenetics and Nutrition Research, Athens Euroclinic Hospital, 11521 Athens, Greece; en.ygeia@gmail.com; 2 Hellenic Institute of Nutrition, 15125 Athens, Greece; 3 Department of Nutritional Sciences and Dietetics, School of Health Sciences, International Hellenic University, 57400 Thessaloniki, Greece; 4 Department of Clinical Sciences and Community Health, Università degli Studi di Milano, 20122 Milan, Italy; 5 Department of Life Sciences, School of Life and Health Sciences, University of Nicosia, CY-2417 Nicosia, Cyprus; 6 Pediatric Unit, Fondazione IRCCS Ca’ Granda-Ospedale Maggiore Policlinico, 20122 Milan, Italy

**Keywords:** berberine, *Morus alba*, 1-deoxynojirimycin, chromium picolinate, insulin resistance, Matsuda index, obesity, randomized controlled trial, appetite, metabolic health

## Abstract

Objectives: To evaluate the efficacy of a fixed-dose combination of berberine, white mulberry leaf extract, and chromium picolinate on body weight, body composition, and insulin sensitivity in Greek adults with obesity. Research Methodology/Design: This was a 12-week, randomized, double-blind, placebo-controlled trial. Adults with obesity and no diagnosed chronic disease were randomly assigned to receive either berberine (1000 milligrams), white mulberry leaf extract (400 milligrams standardized to 5% 1-deoxynojirimycin), and chromium picolinate (0.644 milligrams) or a matching placebo, administered in two daily doses before the main meals. Setting: The study was conducted in Greece among community-dwelling adults with obesity. Main Outcome Measures: The primary outcome was insulin sensitivity, assessed using the Matsuda Composite Insulin Sensitivity Index derived from a 75-g oral glucose tolerance test. Secondary outcomes included body mass index, total body fat mass, and visceral fat rating. Results: Ninety-three participants were randomized, with 46 assigned to the active intervention and 47 to placebo. Compared with placebo, the active intervention produced significantly greater reductions in body mass index (adjusted mean difference −1.24 kg per square meter; 95% confidence interval −2.15 to −0.33), total body fat mass (−2.35 kg; 95% confidence interval −4.12 to −0.58), and visceral fat rating (−0.92 units; 95% confidence interval −1.74 to −0.10). Insulin sensitivity improved significantly, with a 28% greater improvement in the active group than in the placebo group (adjusted geometric mean ratio 1.28; 95% confidence interval 1.14 to 1.44). Conclusions: The fixed-dose combination improved BIA-derived body-composition measures and OGTT-derived insulin sensitivity over 12 weeks compared with placebo. Because no single-agent arms were included, individual component contributions and synergy remain undetermined; confirmatory trials using direct metabolic and imaging methods are warranted.

## 1. Introduction

### 1.1. The Epidemiological Burden of Obesity in Greece

Obesity is now recognized worldwide as a chronic, multifactorial disease with serious consequences for public health, quality of life, and the sustainability of health systems. According to the latest data from the World Obesity Federation—Global Obesity Observatory, the obesity rate stands at 12.6% for Greek women and 13.1% for Greek men [1]. Obesity is also a state of chronic, low-grade systemic inflammation that drives insulin resistance and a cluster of related conditions, including type 2 diabetes mellitus (T2DM), hypertension, and non-alcoholic fatty liver disease (NAFLD).

The shift away from the traditional Mediterranean dietary pattern toward a Western-type diet rich in ultra-processed foods, combined with sedentary lifestyles, has contributed to a broader crisis in metabolic health.

### 1.2. Pathophysiological Link Between Visceral Adiposity and Insulin Resistance

Central, or visceral, obesity is considered the principal pathophysiological driver of insulin resistance. Unlike subcutaneous fat, visceral adipose tissue is metabolically highly active. Hypertrophy of visceral adipocytes increases lipolysis and the release of large quantities of free fatty acids (FFAs) into the portal circulation. This increased hepatic FFA flux contributes to the development of hepatic insulin resistance, resulting in increased hepatic glucose production and increased synthesis and secretion of very-low-density lipoprotein (VLDL) [2].

In addition, visceral adipose tissue functions as an endocrine organ, secreting pro-inflammatory cytokines such as interleukin-6 (IL-6) and tumor necrosis factor-alpha (TNF-α), which disrupt insulin signaling in peripheral tissues. This chronic inflammatory status leads to secondary hyperinsulinemia, as the pancreas attempts to compensate for reduced tissue sensitivity by increasing insulin secretion—a process that ultimately leads to β-cell exhaustion and the onset of T2DM [3].

The use of nutraceuticals to improve insulin sensitivity offers an attractive alternative or adjunct to conventional pharmacological treatment, given their generally favorable safety profile and their potential to act synergistically across multiple metabolic pathways. The present randomized controlled trial aimed to investigate the effect of three principal agents on glucose regulation and lipid metabolism: berberine, 1-deoxynojirimycin (DNJ) derived from white mulberry (*Morus alba*) leaf extract, and chromium picolinate.

### 1.3. Berberine: Regulation of AMPK and Hepatic Gluconeogenesis

Berberine, an alkaloid isolated from plants such as Berberis aristata and Coptis chinensis, activates AMP-activated protein kinase (AMPK) [4]. AMPK activation is a critical process for maintaining cellular energy homeostasis, promoting fatty acid oxidation and glucose uptake in skeletal muscle, while simultaneously inhibiting hepatic cholesterol and triglyceride synthesis [5]. At the cellular level, berberine improves insulin sensitivity by increasing insulin receptor expression and promoting translocation of glucose transporter type 4 (GLUT4) to the cell membrane [4]. It has also been shown to reduce expression of genes involved in gluconeogenesis, such as PEPCK and G6Pase, thereby limiting endogenous hepatic glucose production [4]. Table 1 summarizes the principal molecular mechanisms of action of berberine together with their associated metabolic pathways and clinically relevant effects on glucose homeostasis, lipid metabolism, body composition, inflammation, and insulin sensitivity.

Berberine also exerts notable effects in the gut, modifying the composition of the intestinal microbiome [6]. Its capacity to reduce circulating lipopolysaccharide (LPS) endotoxin levels contributes to a reduction in chronic inflammation, which is itself a contributing cause of obesity and metabolic syndrome [6].

### 1.4. White Mulberry (Morus alba) and 1-Deoxynojirimycin (DNJ)

White mulberry leaf extract is rich in 1-deoxynojirimycin (DNJ), a potent and selective inhibitor of intestinal α-glucosidases—a family of enzymes that hydrolyze the α-glycosidic bonds of dietary carbohydrates [7].

The presence of DNJ slows carbohydrate digestion, thereby blunting the sharp postprandial rise in blood glucose (postprandial hyperglycemia) [7]. This, in turn, reduces insulin secretion demand, protecting pancreatic β-cells from overstimulation. Recent research suggests that DNJ may also improve insulin sensitivity through activation of the PI3K/AKT signaling pathway in skeletal muscle, acting synergistically with berberine [8].

The effect of *Morus alba* extract extends to body fat regulation as well, since reduced postprandial insulin secretion limits lipogenesis and favors lipolysis. In addition, DNJ appears to positively influence secretion of the gut hormones GLP-1 and PYY, which contribute to appetite regulation and satiety [9].

### 1.5. Chromium Picolinate: Enhancing Insulin Action

Chromium is an essential trace element that plays a key role in carbohydrate and lipid metabolism. Chromium picolinate is the most bioavailable form of the element and is widely used to improve glycemic control.

The mechanism of action of chromium involves enhancing insulin binding to its receptors and increasing the number of active receptors on the cell membrane [10]. In adipose tissue, this trace element affects cholesterol homeostasis, which in turn improves the functional activity of GLUT4 glucose transporters, particularly under hyperglycemic conditions [10].

## 2. Methods

### 2.1. Study Population and Design

This 12-week, randomized, double-blind, placebo-controlled, parallel-group clinical trial was conducted at Athens Euroclinic Hospital. Recruitment and enrolment occurred from 19 November to 10 December 2024. The intervention began at each participant’s randomization visit, and the overall study was completed on 8 April 2025.

The trial was registered retrospectively in the ISRCTN registry on 15 July 2026 (ISRCTN39970779), approximately 20 months after first enrolment and 15 months after study completion. The prospectively approved protocol specified the eligibility criteria, primary and secondary outcomes, and analysis plan reported here; nevertheless, retrospective registration is acknowledged as a reporting limitation.

**Figure 1:**
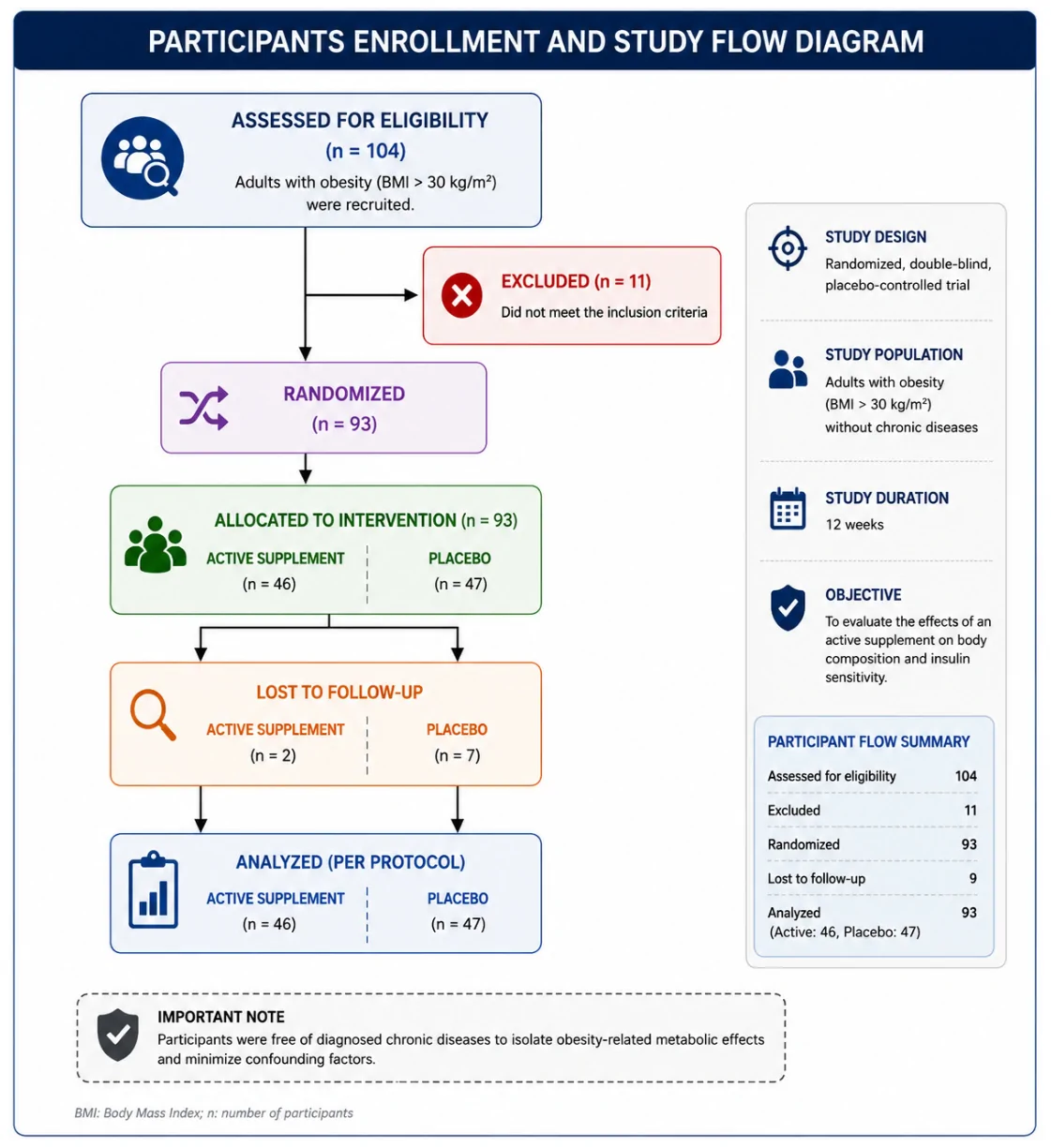
Participant flow diagram.

The participants enrolment flow diagram is presented in Figure 1. A total of 104 adults with obesity (body mass index [BMI] ≥ 30 kg/m^2^) were recruited through advertisements at Athens Euroclinic Hospital and screened for eligibility. Of these, 11 individuals did not meet the inclusion criteria and were excluded. The remaining 93 participants were randomly assigned to receive either the active supplement (n = 46) or a matched placebo (n = 47). During the intervention, two participants in the intervention group and seven participants in the placebo group were lost to follow-up.

Eligible participants were men and women aged 33–68 years with obesity, defined as BMI ≥ 30.0 kg/m^2^ from height and weight measured at screening by trained staff using a calibrated stadiometer and scale, with participants barefoot and in light clothing. Participants were required to have no physician-diagnosed chronic disease, as established through structured medical history, medication review, physical examination, and screening laboratory tests (fasting plasma glucose and insulin, full blood count, ALT, AST, γ-GT, serum creatinine with estimated glomerular filtration rate, lipid profile, and thyroid-stimulating hormone). Hepatic and renal indices had to be within the laboratory reference ranges. Body weight had to be stable within 5% for at least 3 months, and participants had to agree to maintain their habitual diet and physical activity. No glycaemic entry threshold was applied beyond exclusion of pharmacologically treated diabetes.

Exclusion criteria were any physician-diagnosed chronic disease, including type 1 or type 2 diabetes, cardiovascular disease, thyroid disease, inflammatory bowel disease, or malignancy; pregnancy, breastfeeding, or intention to conceive; significant hepatic impairment or renal insufficiency; previous or planned bariatric surgery; known hypersensitivity to berberine, mulberry, or chromium-containing compounds; current anticoagulant or antiplatelet therapy; use of weight-loss, glucose-lowering, or insulin-sensitizing medication or supplements within 4 weeks of screening; use of systemic corticosteroids, antipsychotics, or other agents known to affect body weight or glucose metabolism within 3 months; or participation in another interventional trial within 3 months.

### 2.2. Rationale for Dosing and Study Design

The active product was a fixed-dose combination in an oral hydroxypropyl methylcellulose (HPMC) capsule. All three active components were contained in the same capsule and were not administered separately. Each capsule contained berberine hydrochloride 500 mg (≥97% purity; equivalent to approximately 452.5 mg berberine), *Morus alba* leaf extract 200 mg standardized to 5% 1-deoxynojirimycin (10 mg DNJ), chromium picolinate 0.322 mg (40 μg elemental chromium), magnesium stearate, and precipitated silica. Participants took one capsule twice daily, 15–30 min before lunch and dinner with water, for 12 consecutive weeks. The daily dose was therefore berberine hydrochloride 1000 mg (approximately 905 mg berberine), *Morus alba* leaf extract 400 mg providing 20 mg DNJ, and chromium picolinate 0.644 mg providing 80 μg elemental chromium. The placebo, manufactured by the same producer, contained microcrystalline cellulose and was identical in size, shape, colour, weight, odour, taste, packaging, and randomization coding. Products were stored below 25 °C and protected from light. Dose selection was based on the clinical efficacy and safety literature [4,6,7,10].

*Participants received the following total daily amounts:*

- Berberine hydrochloride: 1000 mg (approximately 905 mg berberine).
- White mulberry leaf extract: 400 mg, standardized to 5% DNJ (20 mg 1-deoxynojirimycin).
- Chromium picolinate: 0.644 mg (80 μg elemental chromium).

### 2.3. Body-Composition Assessment

Body composition, including total body fat mass and visceral fat rating, was assessed at baseline and Week 12 using a Tanita MC-980 Plus multi-frequency BIA analyser (Amsterdam, The Nederlands). Assessments were conducted after an overnight fast of at least 8 h, after voiding, before the OGTT, at the same time of day, with participants barefoot and in light clothing, after abstention from alcohol for 24 h and vigorous exercise for 12 h. The same device and operator were used throughout. The visceral fat rating is a dimensionless whole-unit ordinal index (1–59) derived from a proprietary algorithm and is a surrogate, not a direct measurement, of visceral adipose tissue area or volume.

### 2.4. Randomization and Lifestyle Tracking Protocols

The 93 eligible participants were randomized in a 1:1 ratio to receive either the active supplement (n = 46) or matching placebo (n = 47) utilizing a computer-generated randomization sequence. The randomization code was generated programmatically by an independent statistician and remained fully masked to both the clinical investigators and the study participants until the final database lock. During follow-up, 2 participants in the active group and 7 participants in the placebo group were lost to follow-up and did not complete the 12-week protocol.

To limit the influence of concurrent lifestyle modification, all participants were instructed to maintain their baseline dietary habits and physical activity throughout the 12-week intervention. Dietary intake was assessed using 3-day food diaries at Weeks 0, 6, and 12 and a validated semi-quantitative Food Frequency Questionnaire at Weeks 0 and 12. Physical activity was assessed using the International Physical Activity Questionnaire at Weeks 0, 6, and 12. Weekly forms documented product adherence and self-reported changes in diet or exercise.

### 2.5. Matsuda Index and OGTT Measurements

Accurate measurement of insulin sensitivity is essential for evaluating the efficacy of interventions in obesity research. The most widely used index, HOMA-IR, is calculated solely from fasting glucose and insulin values. However, HOMA-IR primarily reflects hepatic insulin resistance and does not capture the dynamic tissue response to food intake. In contrast, whole-body insulin sensitivity is better estimated using the Matsuda Composite Insulin Sensitivity Index (ISI-Matsuda) [11].

Whole-body insulin sensitivity was estimated using the modified three-point Matsuda Composite Insulin Sensitivity Index [11]: ISI-Matsuda = 10,000/√[(G_0_ × I_0_) × (Gmean × Imean)], where G_0_ and I_0_ are fasting plasma glucose and serum insulin, respectively, and Gmean = (G_0_ + G_60_ + G_120_)/3 and Imean = (I_0_ + I_60_ + I_120_)/3 during a 75 g OGTT. No rescaling was applied. Higher values indicate greater insulin sensitivity. The original Matsuda index uses five OGTT time points; therefore, values from this abbreviated protocol are not numerically identical to five-point estimates.

### 2.6. Statistical Analysis

Continuous variables are presented as mean ± standard deviation (SD) unless otherwise specified. Prior to inferential analysis, the assumptions for parametric testing were evaluated. Normality of distribution was assessed using the Shapiro–Wilk test, and homogeneity of variances was examined using Levene’s test. Normality and homogeneity assumptions were considered acceptable for the anthropometric and body-composition outcomes. Because the Matsuda index exhibited positive skewness, analyses for this endpoint were conducted on the log-transformed scale.

To evaluate the efficacy of the active supplement relative to placebo over the 12-week intervention period, analysis of covariance (ANCOVA) was performed for each clinical endpoint. For each model, the Week 12 value of the outcome was entered as the dependent variable, treatment group (active supplement vs. placebo) as the fixed factor, and the corresponding baseline value as the covariate. This approach was used to account for baseline differences between groups and to estimate the adjusted treatment effect at Week 12.

For the Matsuda index, ANCOVA was performed on log-transformed Week 12 values, with the log-transformed baseline Matsuda value included as a covariate. Results for this endpoint are therefore presented as geometric means at baseline, and as geometric mean ratios (GMRs) for adjusted between-group comparisons following back-transformation.

All efficacy analyses followed the intention-to-treat principle. Week-12 data were observed for 44 active-group and 40 placebo-group participants; missing outcome data were handled using multiple imputation. Within-group baseline-to-Week-12 changes were estimated for each arm, while the prespecified treatment effects were assessed by baseline-adjusted ANCOVA. Statistical significance was defined as a two-sided *p* < 0.05. Nominal model-based *p* values (Punadj) and multiplicity-adjusted *p* values (Padj) were reported across the prespecified efficacy endpoints, together with partial eta-squared (η^2^p). Analyses were performed using IBM SPSS Statistics (version 31.0). No formal a priori sample-size calculation was conducted; enrolment was feasibility-based. Using the achieved allocation (46 and 47 participants) and residual variability from the ANCOVA models, the minimum detectable between-group effects at 80% power and two-sided α = 0.05 were a Matsuda geometric mean ratio of 1.18, 1.30 kg/m^2^ for BMI, 2.53 kg for total body fat mass, and 1.17 units for visceral fat rating. Secondary endpoints, particularly visceral fat rating, should therefore be interpreted as supportive or exploratory.

## 3. Results

### 3.1. Baseline Characteristics and Behavioral Stability

Baseline demographic, anthropometric, and biochemical markers did not differ significantly between the active supplement and placebo groups (*p* > 0.05), consistent with adequate comparability between groups at baseline. The baseline values confirm that the cohort presented with marked obesity, central adiposity, and metabolic impairment, as evidenced by elevated visceral fat ratings and suppressed Matsuda Composite Insulin Sensitivity Index scores (Table 2).

Self-reported energy intake remained stable over 12 weeks in both groups, with no evidence of a differential change between groups. At baseline, daily energy intake was 2155 ± 284 kcal/day in the active group and 2178 ± 293 kcal/day in the placebo group (mean difference −23 kcal/day, 95% CI −142 to 96; *p* = 0.70).

Physical activity also remained stable. Baseline values were 1124 ± 185 and 1158 ± 192 MET-min/week in the active and placebo groups, respectively (mean difference −34 MET-min/week, 95% CI −112 to 44; *p* = 0.39) (Table 3).

Taken together, these findings indicate that self-reported energy intake and physical activity remained stable in both arms, with no evidence of differential change. Because food records, FFQs, and the IPAQ are self-reported and susceptible to misreporting and reactivity, these procedures reduce, but do not eliminate, the possibility that unmeasured lifestyle changes contributed to the observed effects.

### 3.2. Week 12 Efficacy Outcomes

#### 3.2.1. Body Mass Index (BMI)

Baseline BMI was 33.15 ± 2.45 kg/m^2^ in the active supplement group and 34.22 ± 2.38 kg/m^2^ in the placebo group. At Week 12, ANCOVA adjusted for baseline BMI demonstrated a statistically significant treatment effect in favor of the active supplement, with an adjusted mean difference of −1.24 kg/m^2^ (95% CI: −2.15 to −0.33; P_unadj_ = 0.008; P_adj_ = 0.024). The corresponding effect size was η^2^p = 0.11, indicating a moderate treatment effect.

#### 3.2.2. Total Body Fat Mass

Baseline total body fat mass was 35.40 ± 4.85 kg in the active supplement group and 36.95 ± 5.12 kg in the placebo group. ANCOVA adjusting for baseline fat mass showed a significant between-group difference at Week 12, favoring the active supplement, with an adjusted mean difference of −2.35 kg (95% CI: −4.12 to −0.58; P_unadj_ = 0.010; P_adj_ = 0.024). The corresponding effect size was η^2^p = 0.09, consistent with a moderate treatment effect.

#### 3.2.3. Visceral Fat

**Figure 2:**
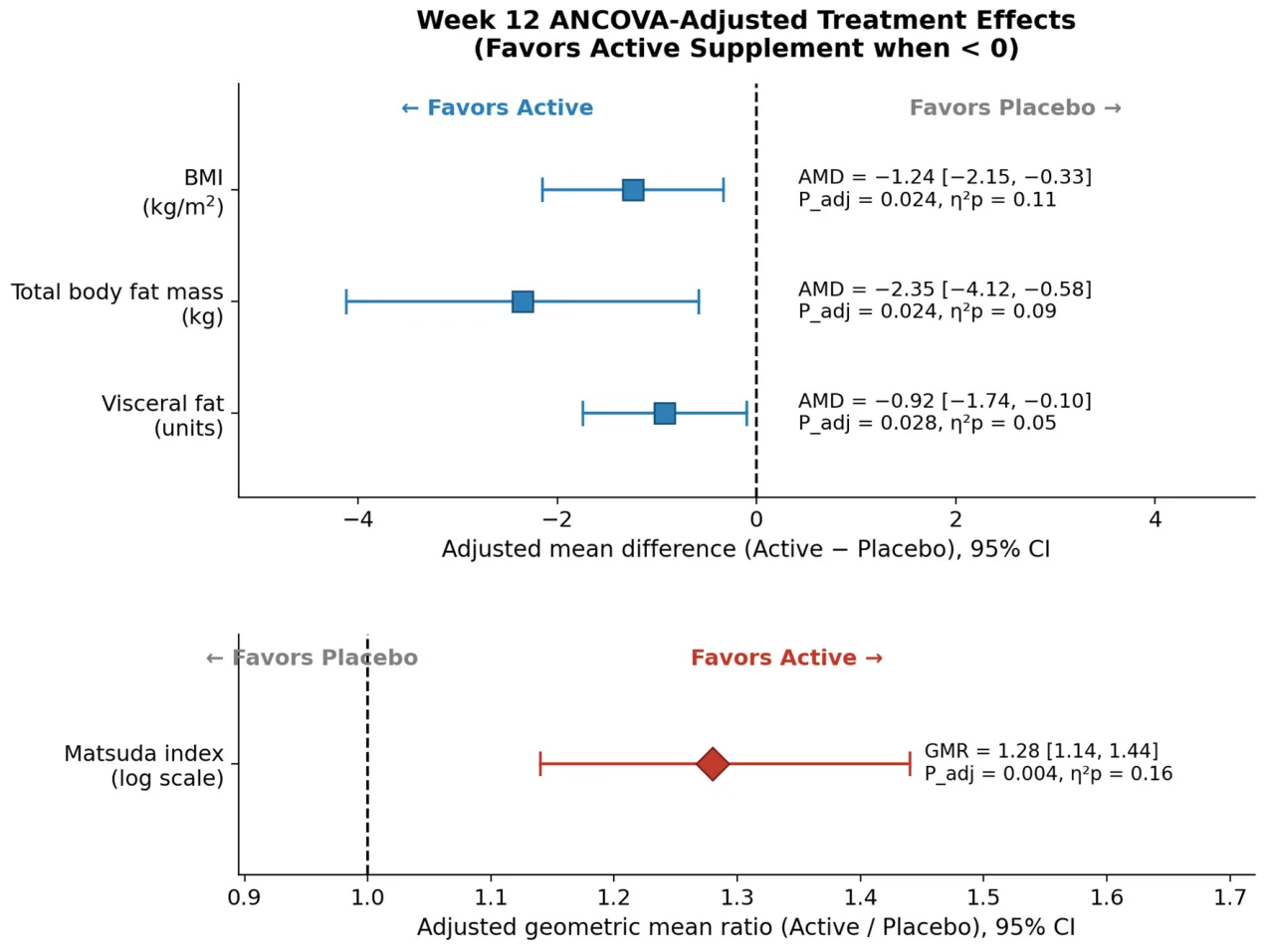
ANCOVA-Adjusted Treatment Effects on Body Composition and Insulin Sensitivity after 12 Weeks of Supplementation. Squares in the upper panel are ANCOVA-adjusted mean differences (active − placebo) and the diamond in the lower panel is the adjusted geometric mean ratio (active/placebo); horizontal bars are 95% confidence intervals, and the dashed vertical line marks the line of no effect (0 for adjusted mean differences and 1.0 for the geometric mean ratio). The coloured left- and right-pointing arrows indicate the direction of benefit on each axis: in the upper panel, values to the left of 0 favour the active supplement, whereas in the lower panel, values to the right of 1.0 favour the active supplement. Arrow colour corresponds to the effect marker of the same panel (blue for adjusted mean differences and red for the geometric mean ratio), and grey denotes the direction favouring placebo. AMD, adjusted mean difference; GMR, geometric mean ratio; Padj, multiplicity-adjusted *p* value; η^2^p, partial eta-squared.

Baseline visceral fat ratings were 13.6 ± 2.2 units in the active supplement group and 14.8 ± 2.5 units in the placebo group. After adjustment for baseline visceral fat, ANCOVA indicated a statistically significant between-group difference at Week 12, with the active supplement group showing lower values than the placebo group (Figure 2). The adjusted mean difference was −0.92 units (95% CI: −1.74 to −0.10; P_unadj_ = 0.028; P_adj_ = 0.028). The corresponding effect size was η^2^p = 0.05, indicating a small-to-moderate treatment effect.

#### 3.2.4. Matsuda Metabolic Index

Because the Matsuda index exhibited positive skewness, values were analyzed on the log-transformed scale, and baseline values are presented as geometric means. Baseline Matsuda index values were 2.38 (95% CI: 1.95 to 2.90) in the active supplement group and 2.62 (95% CI: 2.12 to 3.24) in the placebo group. ANCOVA performed on log-transformed Week 12 Matsuda values, with log-transformed baseline Matsuda index included as a covariate, demonstrated a statistically significant treatment effect. Exponentiation of the treatment coefficient yielded an adjusted geometric mean ratio (GMR) of 1.28 (95% CI: 1.14 to 1.44; P_unadj_ < 0.001; P_adj_ = 0.004), indicating a 28% higher Matsuda index in the active supplement group relative to placebo at Week 12, after baseline adjustment. The corresponding effect size was η^2^p = 0.16, indicating a moderate-to-large treatment effect (Table 4). Plasma glucose and serum insulin concentrations at each time point of the 75 g oral glucose tolerance test, at baseline and at Week 12, are presented in Table 5.

### 3.3. Safety, Tolerability, and Clinical Adverse Events

The active combination demonstrated a favorable safety profile over the 12-week intervention. No serious adverse events or clinically significant laboratory abnormalities were observed in either study arm. Mild, transient gastrointestinal symptoms (including abdominal distension, nausea, and flatulence) were the only treatment-related adverse events reported in the active group and led to discontinuation in two participants (4.3%). Overall, study attrition was higher in the placebo group than in the active-treatment group (7 vs. 2 participants, respectively). No additional safety concerns were identified during follow-up.

## 4. Discussion

### 4.1. Overview

In this 12-week, randomized, double-blind, placebo-controlled trial, the active combination of berberine, white mulberry leaf extract and chromium picolinate produced significantly greater improvements than placebo across all pre-specified efficacy endpoints, body-composition and insulin-sensitivity outcomes.

### 4.2. Changes in BMI and Body Composition: Interpretation and Clinical Significance

The reduction in BMI was accompanied by a reduction in total body fat mass measured by bioelectrical impedance analysis (BIA). The adjusted between-group differences in BMI (−1.24 kg/m^2^) and BIA-derived fat mass (−2.35 kg) numerically exceed pooled estimates reported for berberine monotherapy; however, cross-trial comparisons cannot establish superiority of the combination or identify the contribution of individual components [12,13].

Accordingly, the present findings establish an effect of the fixed-dose formulation relative to placebo but do not show incremental benefit from white mulberry extract or chromium picolinate beyond berberine monotherapy. A 2026 multicentre trial found no significant effect of six months of berberine monotherapy on imaging-measured visceral adipose tissue or liver fat in diabetes-free adults with obesity and metabolic dysfunction-associated steatotic liver disease [14]. That outcome is not directly comparable with the present BIA-derived visceral fat rating: imaging provides an anatomical measurement in absolute units, whereas the Tanita rating is a dimensionless ordinal index generated by a proprietary algorithm. Differences between the studies therefore cannot be attributed to the additional formulation components.

We therefore make no mechanistic claim about anatomical visceral adiposity. The reduction in BIA-derived visceral fat rating is best interpreted as an exploratory, directionally consistent signal of reduced central adiposity, a phenotype whose reduction has been associated with improvement in cardiometabolic markers and with lower residual cardiovascular risk [15,16]. The index is reported in whole units, and the adjusted difference of 0.92 units is smaller than the resolution of an individual reading and below the effect the trial was powered to detect. Whether the intervention reduces anatomically measured visceral adipose tissue requires confirmation by computed tomography, magnetic resonance imaging, or dual-energy X-ray absorptiometry.

### 4.3. Improvement in Insulin Sensitivity: Interpretation in the Context of Existing Evidence

The Week-12 increase in the Matsuda index (adjusted geometric mean ratio 1.28 versus placebo) indicates improved OGTT-derived insulin sensitivity but does not, by itself, prove improved intrinsic tissue-level insulin action.

The white mulberry leaf component offers a complementary and mechanistically distinct route to positive outcome. A 2026 meta-analysis of 23 randomized trials found that mulberry leaf extract reduced fasting glucose by 0.72 mmol/L, postprandial glucose by 1.05 mmol/L, and HbA1c by 0.31% in individuals with type 2 diabetes or prediabetes [17]. Because the Matsuda index is calculated from glucose and insulin values obtained during an oral glucose tolerance test, a treatment effect exerted primarily on postprandial glucose excursions—as would be expected from DNJ-mediated α-glucosidase inhibition—could contribute directly to an apparent improvement in this index without necessarily reflecting a change in intrinsic tissue-level insulin sensitivity.

A decomposition of ln(ISI-Matsuda) indicated that approximately 40% of the treatment effect arose from the fasting glucose–insulin component and approximately 60% from the dynamic post-load component. The latter is compatible with DNJ-mediated slowing of carbohydrate digestion, but it also incorporates post-load insulin concentrations and therefore cannot quantify absorption independently of β-cell secretion and tissue insulin action. The fasting component is consistent with improved predominantly hepatic insulin sensitivity, although an indirect effect of sustained reductions in postprandial glucotoxicity cannot be excluded. Definitive attribution requires an insulin-sensitivity method independent of enteral glucose absorption, such as a hyperinsulinaemic–euglycaemic clamp.

### 4.4. Proposed Complementary Mechanisms of Action

The formulation components plausibly act at different points in glucose handling. DNJ inhibits intestinal α-glucosidase and slows carbohydrate digestion [7,9]; berberine may influence hepatic and skeletal-muscle pathways through AMPK, GLUT4, fatty-acid oxidation, and gluconeogenic gene expression [4,5,18]; and chromium picolinate may modulate insulin-receptor signalling [10,19]. These mechanisms provide a hypothesis for a combined effect, not evidence of synergy. Because the trial compared one fixed-dose combination with placebo, an additive effect, a synergistic interaction, and an effect driven by one dominant component are not distinguishable. Synergism would require independent variation of components in a factorial design and formal interaction testing.

### 4.5. Strengths

Several features of the study design strengthen confidence in the reported findings:

Randomized, double-blind, placebo-controlled parallel-group design, with a computer-generated allocation sequence held by an independent statistician and concealed from investigators and participants until database lock, minimizing selection and detection bias.

Assessment of BMI together with BIA-derived total body fat mass and visceral fat rating provided complementary anthropometric information, although BIA cannot fully distinguish adipose change from hydration-related measurement variation. The modified three-point Matsuda index also incorporated both fasting and post-load glucose–insulin responses, while remaining sensitive to changes in enteral glucose absorption.

Multi-method behavioural monitoring—3-day food diaries, a validated Food Frequency Questionnaire, IPAQ assessment, and weekly compliance forms—showed no differential self-reported lifestyle change between groups. These measures reduce, but cannot eliminate, residual confounding from unmeasured or misreported behavioural changes.

Finally, intention-to-treat analysis with multiple imputation for missing outcome data and multiplicity adjustment across prespecified endpoints, reducing the risk of inflated or biased effect estimates.

### 4.6. Limitations

Several limitations should be acknowledged. First, this single-centre trial had a modest, feasibility-based sample (n = 93), limiting precision for secondary endpoints and generalizability. Second, the trial was retrospectively registered; although the protocol and outcomes had ethics approval before enrolment, prospective registration was not completed. Third, the fixed-dose design included no single-agent or factorial arms; individual contributions, additivity, synergy, and superiority to monotherapy cannot be determined. Fourth, attrition differed between arms (4.3% versus 14.9%), which may introduce bias despite intention-to-treat analysis and multiple imputation. Fifth, body composition was assessed by BIA. BIA-derived fat mass is sensitive to hydration and body geometry, while the visceral fat rating is a proprietary whole-unit ordinal index rather than an anatomical measurement. The visceral-fat result is exploratory and requires imaging confirmation. Sixth, self-reported dietary intake and physical activity cannot exclude residual behavioural change. Finally, no post-intervention follow-up was performed: Week 12 coincided with the final supplementation day, so durability or rebound after discontinuation is unknown. Because DNJ inhibition is competitive and meal-dependent, its postprandial effect may not persist after the last dose. Withdrawal or extension studies are needed.

### 4.7. Differential Attrition Between Study Groups

An additional observation was the higher attrition rate in the placebo group compared with the active-treatment group (14.9% vs. 4.3%). Although participant interviews did not indicate that treatment allocation had been correctly identified or that study blinding had been compromised, differential attrition is a well-recognized challenge in obesity trials. Previous systematic reviews have shown that placebo-treated participants frequently exhibit higher non-adverse-event dropout rates than those receiving active pharmacological interventions. This phenomenon has been attributed to the combination of high expectations for weight loss, the absence of perceived clinical benefit, and the cumulative burden of prolonged protocol requirements, including repeated clinic visits, behavioral monitoring, and dietary recording. Consequently, reduced motivation rather than treatment unblinding is considered a common explanation for differential retention in placebo-controlled obesity studies [20,21].

## 5. Conclusions and Future Directions

This randomized, double-blind, placebo-controlled trial found that 12 weeks of the fixed-dose combination was associated with improved BIA-derived body-composition measures and OGTT-derived insulin sensitivity relative to placebo in adults with obesity. These findings apply to the formulation as a whole. Because there were no single-agent comparators, the contribution of each component and the presence or absence of synergy remain undetermined. Larger prospectively registered trials using direct measures of insulin sensitivity and imaging-based adiposity outcomes are required before clinical or mechanistic conclusions can be drawn.

Future studies should examine durability during treatment and after withdrawal, isolate individual and combined component effects using factorial designs, confirm adiposity outcomes by imaging, and assess insulin sensitivity with methods independent of enteral glucose absorption. Extension assessments at 4, 12, and 24 weeks after discontinuation would help determine whether effects persist or rebound. Studies in populations such as type 2 diabetes or polycystic ovary syndrome may also be informative [22].

## Figures and Tables

**Table 1:** Mechanisms of action of berberine and corresponding metabolic and clinical effects [4].

Mechanism of Action	Metabolic Effect	Clinical Benefit
AMPK activation	Increased fatty acid oxidation	Reduced body fat and NAFLD
GLUT4 translocation	Enhanced muscle glucose uptake	Improved insulin sensitivity
Inhibition of gluconeogenesis	Reduced hepatic glucose output	Reduced fasting glucose and HbA1c
Gut microbiome modulation	Reduced Firmicutes/Bacteroidetes ratio	Reduced systemic inflammation

**Table 2:** Study population’s baseline characteristics.

Baseline Parameter	Active Group (n = 46)	Placebo Group (n = 47)	Between-Group Difference (*p* Value) *
Age (years)	44.3 ± 8.1	45.1 ± 7.9	0.632
Sex (Male/Female)	18/28	17/30	0.771
Fasting Blood Glucose (mg/dL)	98.6 ± 7.4	97.9 ± 8.1	0.665
Fasting Insulin (μIU/mL)	14.2 ± 3.5	13.8 ± 3.2	0.571
Body Mass Index (kg/m^2^)	33.15 ± 2.45	34.22 ± 2.38	0.165
Total Body Fat Mass (kg)	35.4 ± 4.85	36.95 ± 5.12	0.138
Visceral Fat Rating (units)	13.6 ± 2.2	14.8 ± 2.5	0.052

* *p* values refer to the between-group comparison at baseline: independent-samples *t*-test for continuous variables and Pearson’s chi-squared test for sex. Continuous variables are presented as mean ± standard deviation.

**Table 3:** Comparative Analysis of Dietary Intake and Physical Activity Parameters (Behavioral Stability).

Parameter	Active Baseline	Active Week 12 *	Placebo Baseline	Placebo Week 12 *	Baseline Difference (95% CI); *p*	Week-12 Between-Group *p*
Daily energy intake (kcal/day)	2155 ± 284	2138 ± 271	2178 ± 293	2182 ± 280	−23 (−142 to 96); 0.70	0.842
Physical activity (MET-min/week)	1124 ± 185	1140 ± 179	1158 ± 192	1145 ± 188	−34 (−112 to 44); 0.39	0.912
Matsuda index †	2.38 (1.95–2.90)	3.06 (2.56–3.66)	2.62 (2.12–3.24)	2.59 (2.10–3.19)	Ratio 0.91 (0.68–1.22); 0.52	—

* Week-12 behavioural values are observed cases (active n = 44; placebo n = 40). † Matsuda values are geometric means (95% CI); Week-12 values are intention-to-treat estimates after multiple imputation. Baseline differences are unadjusted.

**Table 4:** Summary of Week 12 efficacy outcomes (ANCOVA, baseline-adjusted).

Outcome	Active Baseline	Active Week 12	Active Change (95% CI); *p*	Placebo Baseline	Placebo Week 12	Placebo Change (95% CI); *p*	Adjusted Effect (95% CI); Padj
BMI (kg/m^2^)	33.15 ± 2.45	31.94 ± 2.38	−1.21 (−1.58 to −0.84); <0.001	34.22 ± 2.38	34.16 ± 2.41	−0.06 (−0.31 to 0.19); 0.635	−1.24 (−2.15 to −0.33); 0.024
Total body fat mass (kg)	35.40 ± 4.85	33.17 ± 4.65	−2.23 (−2.98 to −1.48); <0.001	36.95 ± 5.12	36.88 ± 5.08	−0.07 (−0.58 to 0.44); 0.785	−2.35 (−4.12 to −0.58); 0.024
Visceral fat rating (units)	13.6 ± 2.2	12.76 ± 2.12	−0.84 (−1.22 to −0.46); <0.001	14.8 ± 2.5	14.70 ± 2.48	−0.10 (−0.38 to 0.18); 0.478	−0.92 (−1.74 to −0.10); 0.028
Matsuda index †	2.38 (1.95–2.90)	3.06 (2.56–3.66)	GMR 1.29 (1.18–1.41); <0.001	2.62 (2.12–3.24)	2.59 (2.10–3.19)	GMR 0.99 (0.92–1.06); 0.742	GMR 1.28 (1.14–1.44); 0.004

† Matsuda values are geometric means (95% CI); changes and adjusted effects are geometric mean ratios (GMRs). Week-12 values are intention-to-treat estimates after multiple imputation. Negative adjusted effects favour active treatment for BMI and adiposity outcomes; GMR > 1 favours active treatment for the Matsuda index.

**Table 5:** Plasma glucose and insulin concentrations during the 75 g oral glucose tolerance test at baseline and Week 12.

Variable	Active Baseline	Active Week 12	Placebo Baseline	Placebo Week 12	Adjusted Effect (95% CI); Padj
Glucose, 0 min (mg/dL)	98.6 ± 7.4	93.4 ± 6.8	97.9 ± 8.1	97.2 ± 7.6	−4.45 (−7.12 to −1.78); 0.008
Glucose, 60 min (mg/dL)	152.4 ± 18.6	131.8 ± 16.2	150.8 ± 19.1	149.5 ± 18.4	−18.82 (−25.40 to −12.24); <0.001
Glucose, 120 min (mg/dL)	126.8 ± 15.2	112.5 ± 13.9	125.1 ± 14.8	124.3 ± 14.5	−12.45 (−17.80 to −7.10); <0.001
Insulin, 0 min (μIU/mL)	14.2 ± 3.5	11.5 ± 2.8	13.8 ± 3.2	13.6 ± 3.1	−2.38 (−3.52 to −1.24); <0.001
Insulin, 60 min (μIU/mL)	168.5 ± 28.4	128.2 ± 24.1	165.2 ± 27.9	163.0 ± 26.5	−36.70 (−46.85 to −26.55); <0.001
Insulin, 120 min (μIU/mL)	116.4 ± 22.1	88.6 ± 18.7	114.8 ± 21.5	112.9 ± 20.8	−25.10 (−32.90 to −17.30); <0.001

## Data Availability

The data presented in this study are not publicly available because the informed consent obtained from the participants did not include provision for public deposition of individual-level data. The de-identified datasets generated and analysed during the study, together with the study protocol and the statistical analysis plan are available from the corresponding author (K.X.) upon reasonable request and subject to approval by the Ethics Committee of Athens Euroclinic Hospital. The trial record is publicly available at https://www.isrctn.com/ISRCTN39970779 (accessed on 15 July 2026).
